# A Model Study to Evaluate Osseointegration and Fracture Healing Following Open Reduction and Internal Fixation (ORIF) in Diabetic Lewis Rats

**DOI:** 10.1053/j.jfas.2023.04.011

**Published:** 2023-05-09

**Authors:** Alexandra Arteaga, Claudia Cristina Biguetti, Bhuvana Lakkasetter Chandrashekar, Jimena Mora, Adeena Qureshi, Evelin Rios, Javier La Fontaine, Danieli C. Rodrigues

**Affiliations:** 1 Department of Bioengineering, The University of Texas at Dallas, Richardson, TX; 2 Department of Surgery and Biomechanics, School of Podiatric Medicine, The University of Texas Rio Grande Valley, Harlingen, TX

**Keywords:** hyperglycemia, implants, orthopedics, rodent, titanium

## Abstract

There is a higher risk of implant osseointegration failure after open reduction and internal fixation (ORIF) in patients with diabetes due to increased inflammatory conditions, associated metallic corrosion and infection. While it is possible to avoid elective osseous surgery in patients with diabetes, it may not be the case in nonelective cases, such as ORIF ankle fractures. A total of 30 male Lewis rats (12–15 weeks old) were distributed into diabetic (D) and nondiabetic (ND) groups. Fracture healing and osseointegration were evaluated at 2-, 10-, and 21-day time points. Microtomographic and histological analysis depicted distinct differences in fracture healing and osseointegration between D and ND animals. Immunohistochemical analysis exhibited elevated proliferation (PCNA) and osteogenic (Runx2) cells for ND animals, while HMGB1 (inflammatory marker) was elevated for D animals during healing. Bone resorption marker CTX-1 was elevated in the plasma of D animals at 2 days, while bone formation marker P1NP was higher for ND animals at 10 days. Overall, this model resulted in delayed implant osseointegration and fracture healing in diabetic animals, highlighting the importance of developing new biomaterials or implant coatings that can improve bone healing outcomes in this patient population.

Titanium (Ti)-based orthopedic devices in fracture fixation are considered effective to restore the function of damaged bones (1,2). Yet, diabetic patients have a higher risk of implant failure due to infection, biomaterial corrosion, lack of signaling molecules, and poor bone healing/vascularization (3–6). Hyperglycemia is associated with increased bone resorption (e.g., CTX-1) and decreased bone formation markers (e. g., P1NP), which can ultimately affect bone quality and healing after surgical procedures (7). The poor bone healing after open reduction internal fixation (ORIF) in patients with diabetes is accentuated in the extremities due to decreased circulation, sensation, and underlying subcutaneous tissue mass. While some minor fractures, such as digital or metatarsal fractures, can be treated conservatively with immobilization with relatively minor consequences, ORIF of malleolar fractures is often necessary, even in the high-risk population.

ORIF procedures are the standard of care for unstable ankle fractures in patients with diabetes (8). However, there are no models simulating these procedures in translational research, which limits the testing of new materials and surface coatings for applications in foot and ankle surgery. Orthopedic fracture models in rodents have been shown to have limitations in translating to human models due to differences in gait patterns, mechanical loading, and intrinsic healing capacity (9). The most commonly used model is the closed midshaft femoral fracture, which is created using a blunt-force guillotine to replicate similar fractures in each animal (10). However, the use of an intramedullary pin or Kirschner wire prior to fracture is invasive and not representative of a clinical scenario, with significant soft tissue damage and a high probability of bone comminution (10). Other bone defect models include the use of drill hole and femoral critical defects, which also have limitations in terms of fracture healing and may not represent endochondral ossification (10). Finally, closed and open fracture surgical models are also complex and challenging to heal, with a high risk of misplaced fractures, limb shortening, or even bone fragment rotation, and a high susceptibility to infection in open fracture models, which is amplified in diabetic animals due to impaired immune systems. Furthermore, it is important to evaluate the kinetics of bone formation, including the expression of proliferation and osteogenic markers around the hardware, and not only in the fracture site. Thus, the goal of this study was to construct a clinically relevant ORIF model to evaluate osseous healing in diabetic Lewis rats. This model allows for new materials or surfaces to be analyzed in a simplistic, reproducible, and cost-effective manner minimizing variability in healing outcomes.

## Materials and Methods

### Animals and Diabetes Induction

A total of 30 male Lewis rats (12–15 weeks old) were equally distributed into diabetic (D) and nondiabetic (ND) groups (IACUC #19–03). The rats were housed at the vivarium throughout the study with access to sterile water and dry food pellets ad libitum. Five (n = 5) animals per group/time point were euthanized at 2-, 10-, and 21-day post-ORIF procedure for collection of tibias and blood serum. For diabetes induction, rats were fed a high-fat diet (Purina LabDiet 5008) at 3 weeks of age for a minimum of 6 weeks prior to administration of streptozotocin (STZ), and continued the high-fat diet until the conclusion of the study. ND rats were fed a standard diet. Rats were fasted for 4 hours prior to STZ (S-0130, Sigma-Aldrich) injection of 55 mg/Kg. ND and D rats were subjected to surgical procedures at 7 days postinjections. Blood was collected from the tail vein to a glucose strip and measured using a glucometer (AlphaTRAK 2 Blood Glucose Monitoring Kit) prior to surgery to verify systemic condition. Rats with ≥250 mg/dL were considered diabetic (11).

### Surgical Protocol for Open Reduction and Fracture Fixation (ORIF)

Prior to surgery, commercially pure titanium (cpTi) screws (0.76 mm × 2 mm StabilokTM, Fairfax Dental Inc., Miami, FL) were used as orthopedic implants. All implants were cleaned by sonicating for 15 minutes each in acetone, DI water, and ethanol solutions. Implants were dried in an oven at 60°C overnight and sterilized in an autoclave. Lewis rats were anesthetized by inhalation of 4% isoflurane followed by injection of ketamine\xylazine (50–100 mg/kg: 20 mg/kg intramuscular). After anesthesia, rats were placed in a left lateral decubitus position. For ORIF procedure, a longitudinal incision was made directly below the knee joint, followed by tibial tuberosity exposure. Muscle divulsion was performed using a periosteal elevator. A longitudinal defect was created on the right tibia (0.1 mm in width, 4 mm in length and 3 mm in depth) using a circular saw (1800020 Fine Science Tools, Foster City, CA) coupled to a surgical micromotor at 800 RPM (NSK Surgic Pro, Hoffman Estates, IL) under constant irrigation with cold saline solution to avoid heating. This defect was created to simulate a standard fracture. Two cpTi threaded dentin screws (0.76 mm × 3.5 mm, Fairfax Dental Inc., Miami, FL) were placed 2 mm apart in a perpendicular to the fracture defect. During surgery, animals received Lidocane HCl/Epinephrine (1:100,000). After surgery, animals received Buprenorphine (0.3 mg/kg, subcutaneous) as analgesia every 12 hours for 72 hours, and Cefazoline (5 mg/kg, intramuscular) as an antibiotic. The muscle and skin layers were sutured using resorbable sutures (5–0 Coated Vicryl Undyed 1 × 27” RB-1). During surgery, animals received Lidocaine HCl/Epinephrine (1:100,000). After surgery, animals received Buprenorphine (0.3 mg/kg, subcutaneous) as analgesia every 12 hours for 72 hours, and Cefazoline (5 mg/kg, intramuscular) as an antibiotic. The muscle and skin layers were sutured using resorbable sutures (5–0 Coated Vicryl Undyed 1 × 27” RB-1). Animals were euthanized by inhalation of 4% isofluorane, followed by injection of ketamin/xylazine (50 mg/kg: 20 mg/kg intraperitoneal), followed by sodium pentobarbital (120 mg/kg, intraperitoneal) overdose. Tibias were harvested and placed in 10% neutral buffered formalin for 24 hours for fixation. Blood serum was collected for ELISA assays.

### Microtomographic Analysis (microCT)

Tibias were imaged using ultra-high-resolution microCT imaging (OI/CT, Milabs). Images were acquired at 50 kV, 0.21 mA, and an exposure time of 75 ms. Projections were reconstructed and converted to DICOM (Digital Imaging and Communications in Medicine) files using PMOD analysis software (PMOD Technologies LLC, Bruker Switzerland AG, Zurich, Switzerland) at a voxel size of 20 *μ*m. Using Imalytics Preclinical (Gremse-IT GmbH, Aachen, Germany), quantification of newly formed bone area (%) in the fracture gap at 21 days was performed.

### Histological Processing and Analysis

After tibias were scanned by microCT, samples were decalcified using 10% ethylenediaminetetraacetic acid (EDTA)-2Na (Sigma, St. Louis, MO), with 2 EDTA changes per week. Samples were cut to 10 mm^2^ sections of tissue surrounding implant areas. Samples were stained with hematoxylin and eosin (H&E) and Goldner Trichrome (GT) with Alcian Blue stain (12). Outcomes of fracture healing and osseointegration were evaluated for bone to implant contact (BIC %), using H&E and GT stain. The best representative section of the 21-day time point was stained with GT and used to measure BIC % as previously described (13,14). In brief, BIC was obtained using Cellsens software (Olympus, Shinjuku City, Tokyo, Japan), to calculate the percentage of bone contact relative to the entire implant length at bone level. Results were presented as mean +/− standard deviation (SD).

### Immunohistochemistry

Immunohistochemistry was used to identify and quantify High Mobility Group Box 1 (HMGB1), proliferating cell nuclear antigen (PCNA), and osteoblasts differentiation (Runx2). Primary antibodies were obtained from Abcam and diluted in the following concentrations: PCNA (ab92552) at 1:1000, Runx2 at 1:500 (ab236639), and 1:400 HMGB1 (ab79823). Samples were prepared, stained and quantified for area density % of each marker using a previously established protocol (15).

### Competitive ELISA Assay for Bone Turnover Makers in Blood Serum

Animals were fasted for 6 hours, and 100 to 200 *μ*L of blood was collected from the heart region. Blood was centrifuged at 10,000 rpm for 3 minutes. The supernatant containing sera was used for evaluation of bone formation (N-terminal propeptide of type 1 procollagen (P1NP), IDS AC-33F1), and bone resorption (C-terminal telopeptide of type I collagen (CTX-1), IDS AC-06F1) using colorimetric ELISA assays (Immunodiagnostic Systems, Tyne and Wear, UK).

### Statistical Analysis

Statistical analysis of blood glucose measurements, MicroCT data, BIC %, and immunohistochemistry were tested for distribution with a Shapiro-Wilk Normality test. Nonparametric data were evaluated using Mann-Whitney nonparametric test. Samples within normality distribution were analyzed using *t*-test. Mann-Whitney or *t*-test was used for comparisons to appraise the significance between time points within a group (nondiabetic 2 days vs nondiabetic 21 days) or between treatments (nondiabetic 21 days vs diabetic 21 days). Statistics was performed using GraphPad Prism 9.0 software (GraphPad Software Inc., San Diego, CA). A *p*-value of 0.05 or lower was considered statistically significant.

## Results

### Clinical Evaluation After ORIF Surgery

**Fig. 1.**
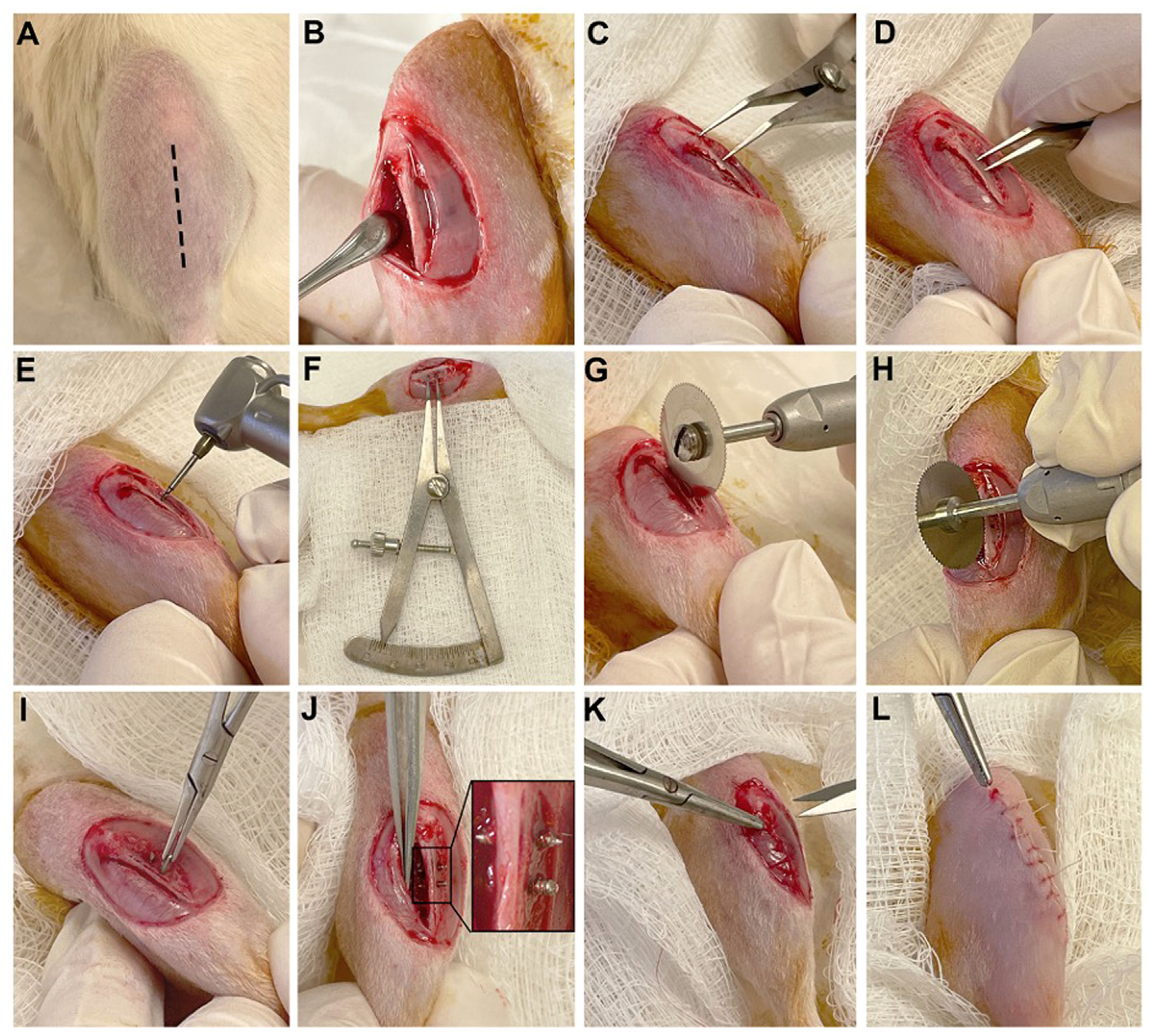
A representative overview of surgical protocol in Lewis rats. (*A*) A vertical incision was performed directly below the knee joint. (*B*) Tibial tuberosity exposure by muscle divulsion using a periosteal elevator. (*C*) An angled 3.25” caliper was used to determine 4 mm distance between knee implant. (*D*) A 2-mm distance between screws was marked using caliper. (*E*) A surgical micromotor at 800 RPM was used to create implant bed. (*F*) Dental Castroviejo caliper was used to verify implant bed distances. (*G, H*) Vertical fracture defect (0.1 mm in width, 4 mm in length and 3 mm in depth) with a circular saw using a surgical micromotor at 800 RPM. (*I, J*) Commercially pure titanium (cpTi) screws (0.76 mm ∅ × 3.5 mm) were placed using microneedle holder perpendicular to fracture defect. (*K*) Muscle layer and (*L*) epidermis layer were sutured.

Animals from ND and D groups were distributed into 3 experimental periods (2, 10, and 21 days) after ORIF procedure (5 animals/group per time point for a total of 30 rats). Surgical protocol is shown in Fig. 1A–L. Both D and ND animals presented no signs of hyperalgesia, with normal behavior considering grooming, eating, and nesting. Postsurgical pain was evaluated as minimal based on the Rat Grimace Scale (16).

### MicroCT and Histological Analysis

**Fig. 2.**
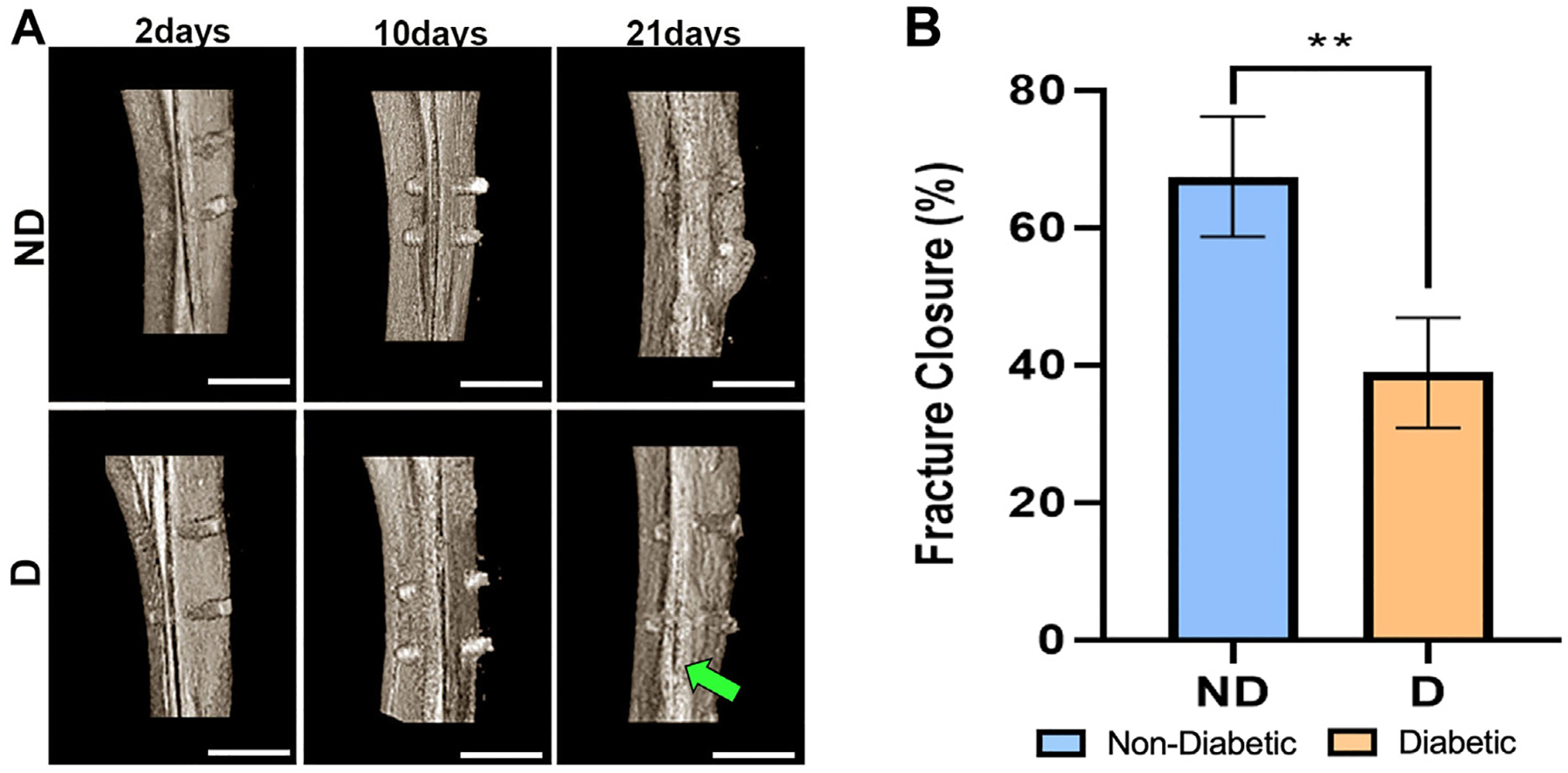
(*A*) Coronal microCT images of tibial implantation sites in ND and D Lewis rats at 2, 10, and 21 days (scale bar = 3.5 mm). Green arrow indicates presence of persistent fracture gap at 21 days. (*B*) Fracture closure was quantified using ImageJ of newly formed bone volume to tissue volume (%) present in the fracture gap at 21 days. Results are presented as means ± SD for bone tissue (%) (n = 5). Symbol ** indicates statistical significance as compared to both ND control groups (*p* ≤ .01).

The microCT analysis revealed the bone quality both at the site of Ti implantation and the fracture site at each time point. As observed in the Fig. 2A, the fracture defect was still present for the D group at 21 days (depicted by the green arrow), while the ND group showed higher signs of fracture closure. Additionally, there was increased mineralized tissue surrounding the implants in the ND group that was not as prevalent in the D group. Fracture healing was quantified at 21 days postimplantation by increased bone hyperdensity in terms of fracture closure (%). ND animals resulted in significantly higher healing (67.43 ± 8.73 fracture closure %) compared to D animals (38.89 ± 7.98 fracture closure %) as seen in Fig. 2B.

**Fig. 3.**
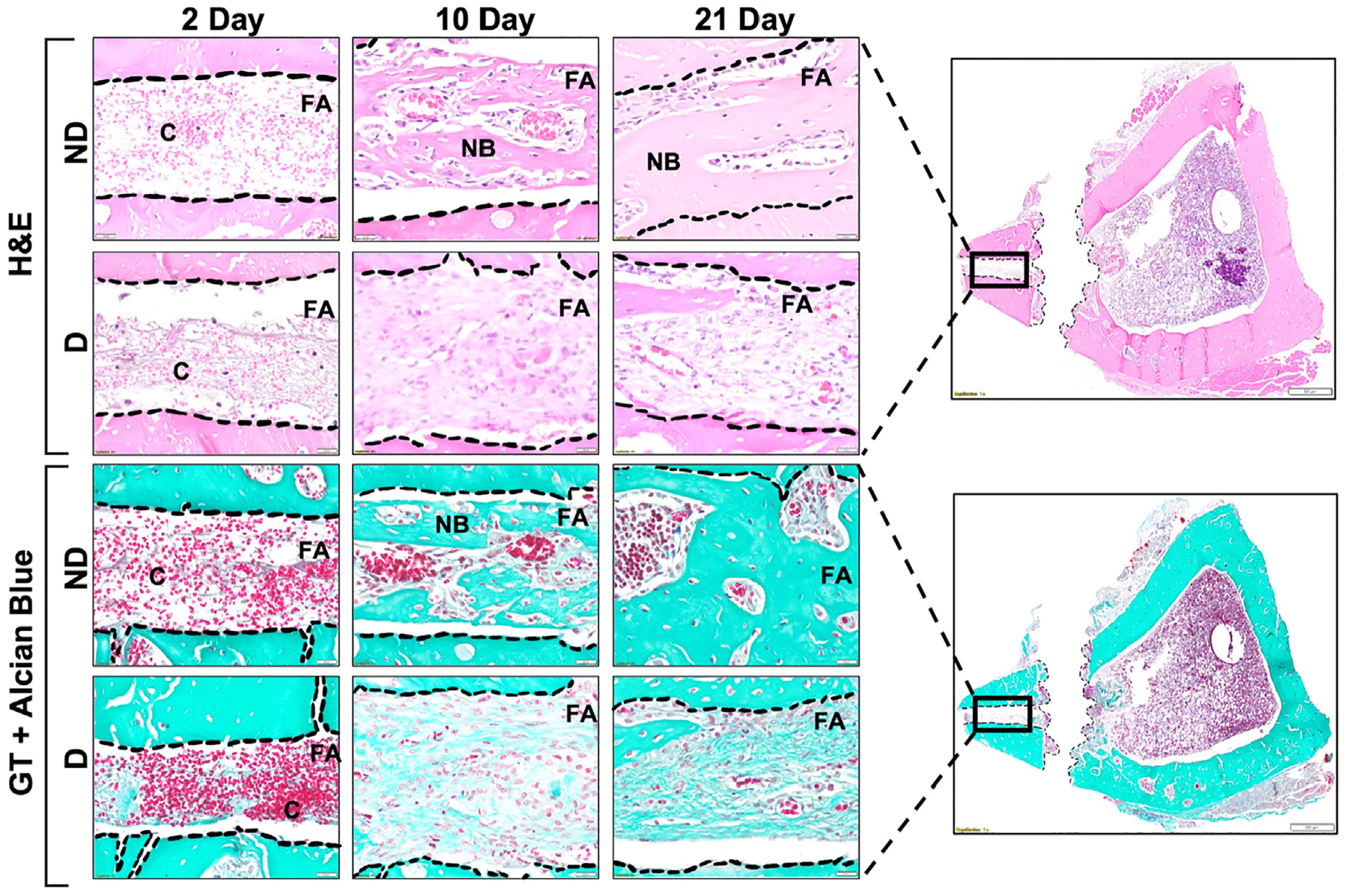
Histological evaluation of fracture sites in ND and D rats at 2, 10, and 21 days after ORIF procedure with panoramic view of a transversal section of a ND rat tibia at 2 days postimplant placement. Fracture area (FA) and blood clots (C) are demonstrated between dashed lines. Scale bar = 20 *μ*m, staining: H&E (top) and GT (bottom), original magnification 40×.

**Fig. 4.**
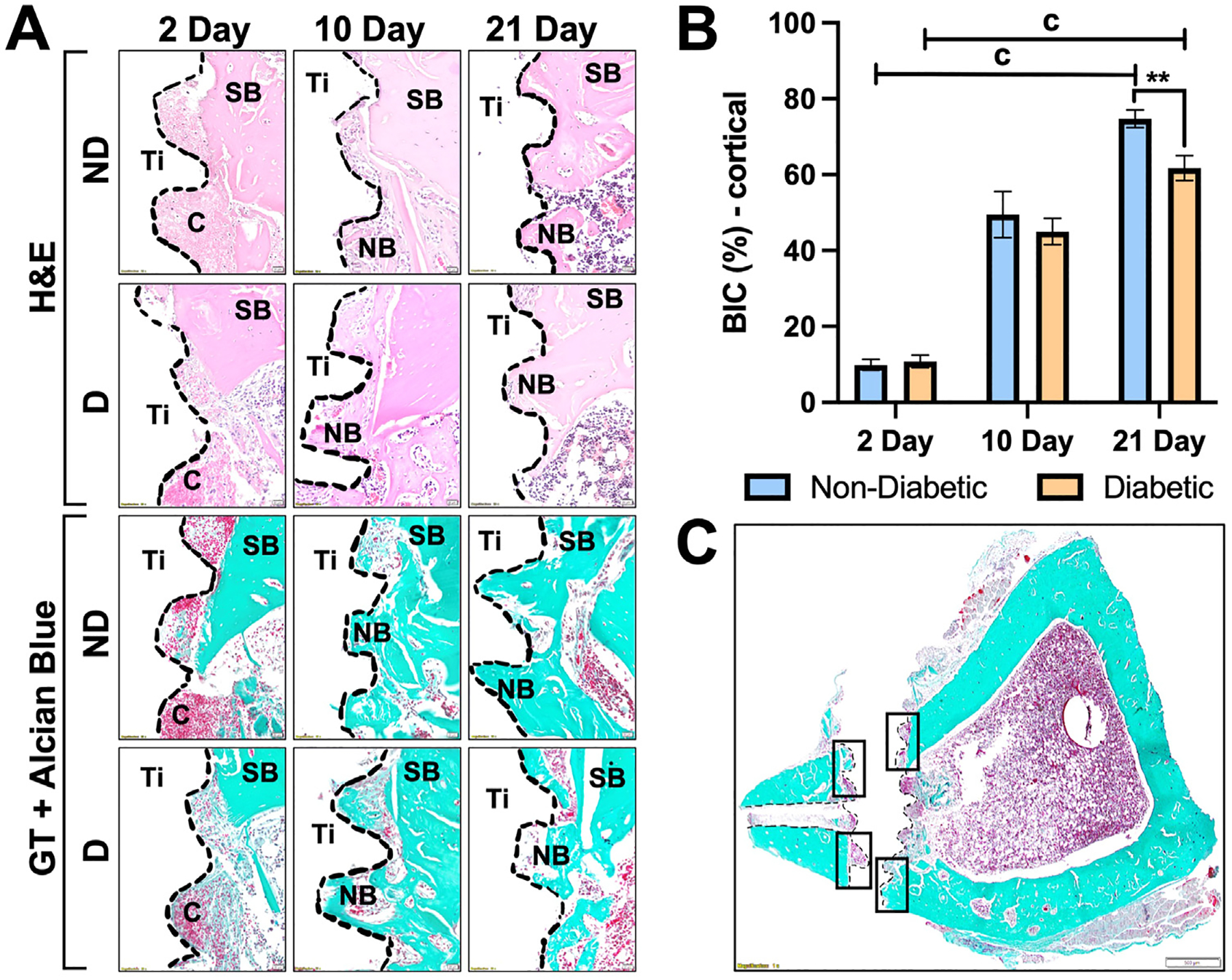
(*A*) Histological evaluation of implantation sites in ND and D rats at 2, 10, and 21 days after implant placement. Implant space (Ti) is demonstrated in the white region between dashed lines, surrounding histological structures include supporting bone (SB), new bone (NB) and blood clot (C). Scale bar = 20 *μ*m, staining: H&E (top) and GT (bottom), original magnification 40×. BIC quantification at 2, 10, and 21 days are shown as means ± SD for BIC (%) of newly formed bone at the implant site (**p ≤ .006) between systemic conditions (D vs ND). (*B*) Significant differences were also observed between time points of D (c: *p* ≤ .007) and ND (c: *p* ≤ .001) groups (n = 5). (*C*) Panoramic view of a transversal section of a ND rat tibia at 2 days postimplant placement demonstrates selected regions for BIC analysis.

Histological evaluation further demonstrated fracture healing (Fig. 3) and osseointegration (Fig. 4A) over time. At 2 days, blood clots were present in both D and ND groups at the fracture and screw areas, which corresponded to hematoma formation and inflammatory events. At 10 days, there was the initiation of new cartilage formation in the fracture area of the ND animals, indicative of callus formation, mineralization, and initial stabilization of the fracture. When compared to the ND animals, the D group resulted in a predominant presence of irregular connective tissues that were rich in fibers and had a diminished presence of blood vessels throughout the fracture at 10 days, resulting in delayed fracture healing at 21 days. At 21 days, the D group resulted in persistent fibrous connective tissue with few areas of new bone in the fracture area, while the ND group showed maturing new bone formation. The quality of new bone formed in ND rats had more mature bone tissue surrounding the implant, while porous bone tissues surrounded the implant surface in both cortical and marrow regions in D rats, shown in Fig. 4A. There was minimal BIC % at 2 days, implant stability was primarily from the supporting bone region for either D (10.82 ± 1.66%) or ND (9.91 ± 1.47%) groups. At 10 days, new bone formation was seen in the screw area of both D (44.99 ± 3.46%) and ND (49.45 ± 6.09%) animals, which was indicative of initial stabilization of bone matrix onto the implant surface. The ND group resulted in significantly higher (*p* ≤ .006) bone formation surrounding the implant (74.68 ± 2.33%) compared to the ND group (61.75 ± 3.31%) at 21 days postimplantation (Fig. 4B). Significant differences were also observed between 2, 10 and 21 days of D (c: *p* ≤ .007) and ND (c: *p* ≤ .001) groups. Selected regions for BIC % are demonstrated in Fig. 4C.

### Immunohistochemical Analysis

**Fig. 5.**
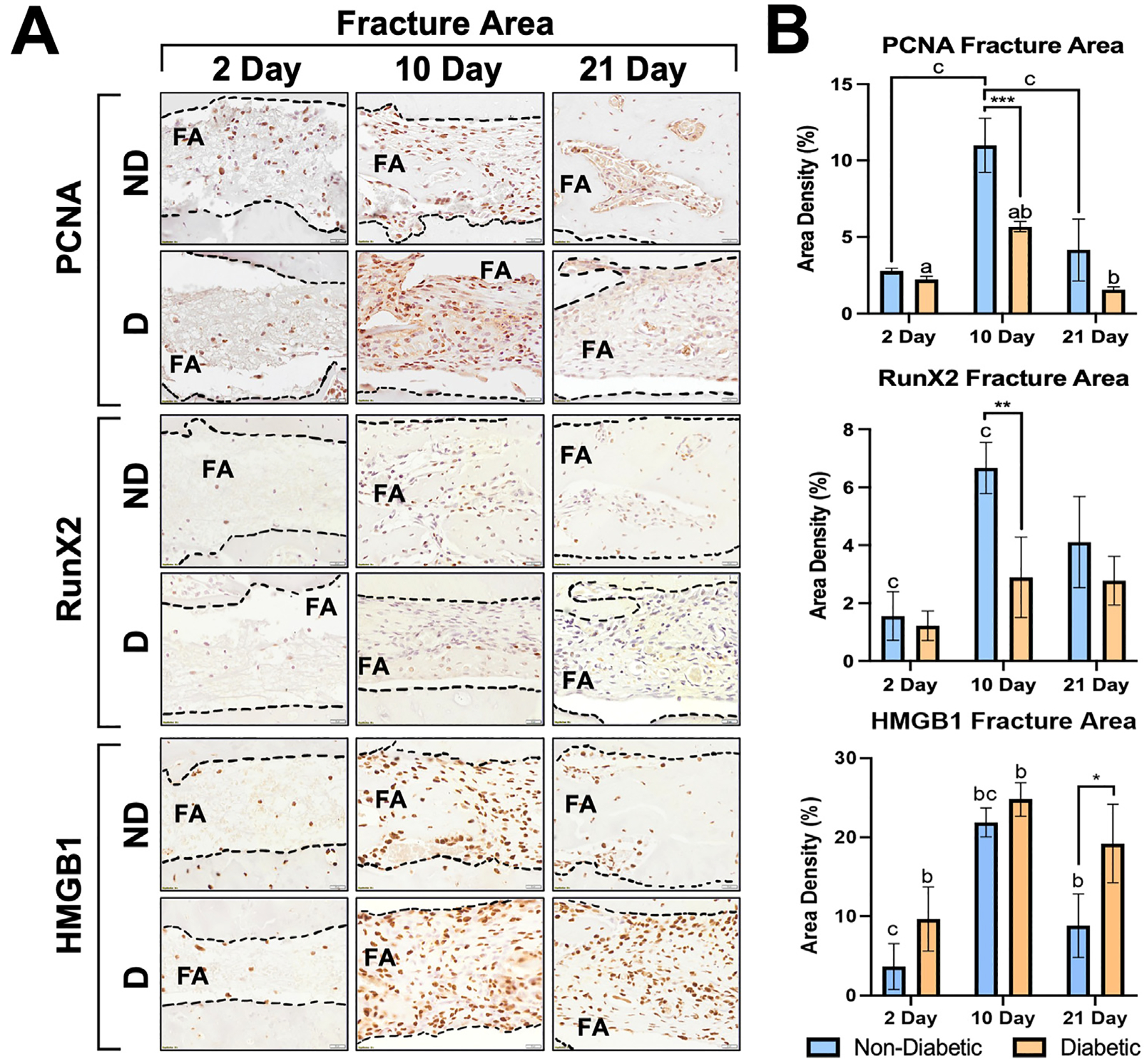
(*A*) Immunohistochemistry for PCNA (top), Runx2 (middle), and HMGB1 (bottom) markers at the fracture sites for D and ND rats at 2, 10, and 21 days. Dark cells: positive labeling. Scale bar: 20*μ*m, original magnification 40×, counterstaining Mayers hematoxylin, chromogen DAB. Quantification of markers identified by immunohistochemical analysis in D and ND rats and are shown as means ±SD for area density (%) at the implant site (*p ≤ .05, **p ≤ .01, ***p ≤ .001) between systemic conditions (D vs ND; n = 5). (B) Significant differences were also observed between time points (a: *p* ≤ .05, b: *p* ≤ .01, and c: *p* ≤ .001). Quantitative results are presented as mean ± SD for each parameter at implants sites during 2, 10, and 21 days.

**Fig. 6.**
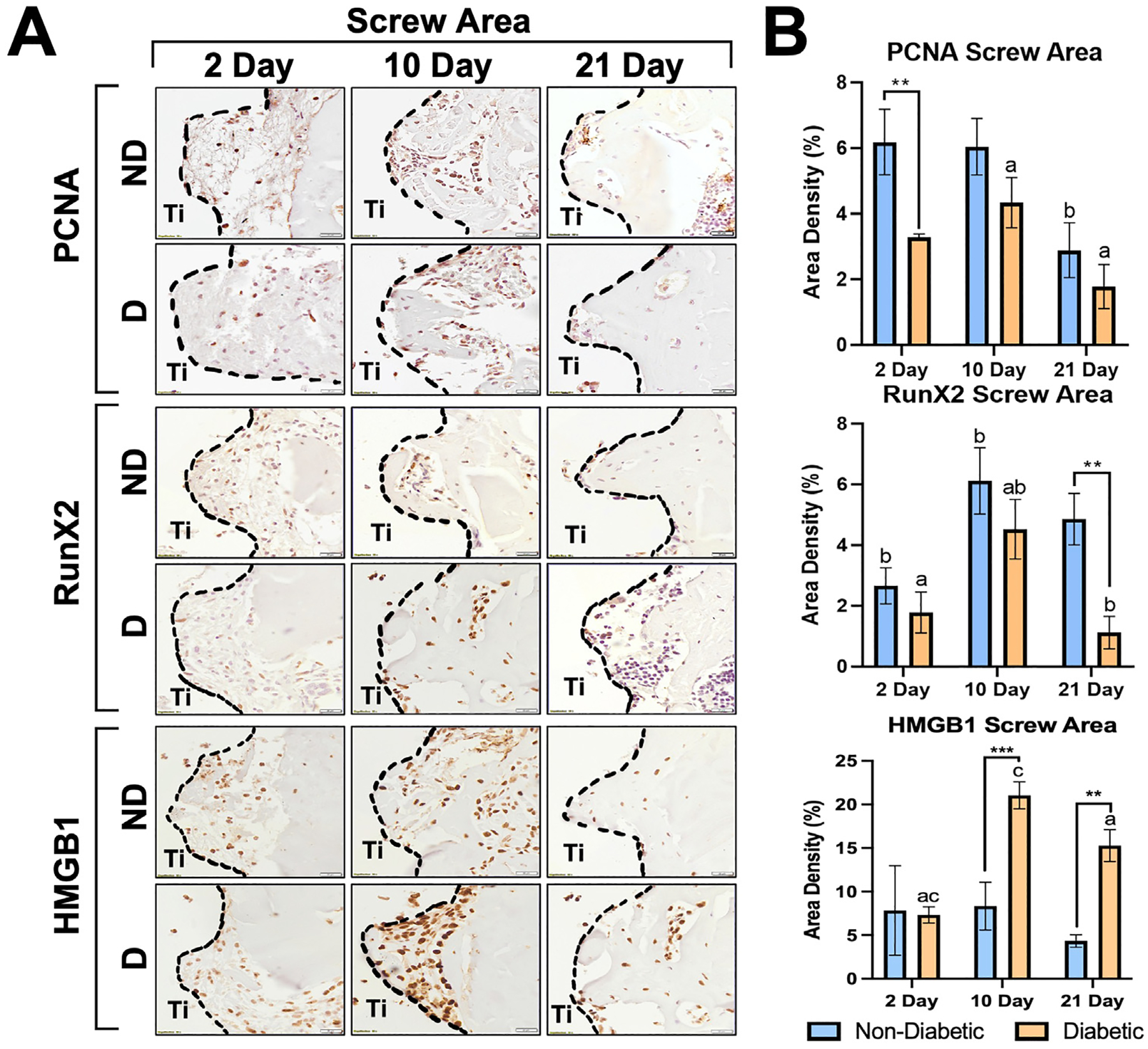
(*A*) Immunohistochemistry for PCNA (top), Runx2 (middle), and HMGB1 (bottom) markers at the implantation sites for D and ND rats at 2, 10, and 21 days. Implant space (Ti) is demonstrated between dashed lines. Dark cells: positive labeling. Scale bar: 20 *μ*m, original magnification 40×, counterstaining Mayers hematoxylin, chromogen DAB. Quantification of markers identified by immunohistochemical analysis in D and ND rats and are shown as means ± SD for area density (%) at the implant site (*p ≤ .05, **p ≤ .01, ***p ≤ .001) between systemic conditions (D vs ND; n = 5). (*B*) Significant differences were also observed between time points (a: *p* ≤ .05, b: *p* ≤ .01, c: and *p* ≤ .001). Quantitative results are presented as mean ± SD for each parameter at implants sites during 2, 10, and 21 days.

Immunohistochemical markers were evaluated for cell proliferation (PCNA+ cells), osteoblast differentiation (Runx2+ cells), and the presence of HMGB1 in fracture healing (Fig. 5A, B) and osseointegration (Fig. 6A, B). For PCNA+ cells, both ND and D animals had significantly higher cell proliferation at 10 days compared to 2 and 21 days within each systemic condition for the fracture area. ND animals resulted in higher proliferation (11.00 ± 1.76%) compared to D animals (5.67 ± 0.33%; *p* < .001). The predominant osteoblast differentiation was also seen in all animals at 10 days but was significantly higher for ND (6.67 ± 0.88%) compared to D animals (2.89 ± 1.39%) as shown in Fig. 5B. A similar trend was depicted by an increase in HMGB1 at 10 days compared to 2 and 21 days, yet D animals conversely had higher HMGB1 present in the fracture area at all time points. Cell proliferation, osteoblast differentiation and HMGB1 receptor markers in fracture area healing over time are demonstrated in Fig. 5A, B.

Considering the tissues surrounding the implant, ND animals had the highest cell proliferation at 2 days, which decreased at 10 days, and appeared to have a significantly lower count at 21 days compared to other time points (*p* ≤ .002). D animals resulted in an increase count of PCNA+ cells at the 10-day time point, with a significant decrease at 21 days (*p* ≤ .014). ND animals resulted in significantly higher proliferation at 2 days (6.19 ± 1.00%) compared to D animals (3.28 ± 0.10%; *p* ≤ .006). The highest osteoblast differentiation was seen in all animals at 10 days. However, the ND animals maintained a high area density % of Runx2 cells in the screw area (4.85 ± 0.85%) while the D animals significantly decreased (1.11 ± 0.54%) at 21 days (*p* ≤ .012). For HMGB1 present in the screw area, ND animals had no significant differences between time points. However, D animals had a significant increase (*p* ≤ .004) of HMGB1 from 2 days (7.33 ± 0.93%) to 10 days (21.06 ± 1.55%) with a marginal decrease at 21 days (15.28 ± 1.84%) that continued to be significantly higher (*p* < .03) than the 2-day time point. Additionally, HMGB1 in D animals was statistically elevated in comparison to ND animals at both 10 (8.33 ± 2.74%, *p* ≤ .007) and 21 days (4.33 ± 0.72%, *p* < .003). Values for cell proliferation, osteoblast differentiation and HMGB1 receptors in tissues surrounding the screw area over time are demonstrated in Fig. 6.

### Bone Turnover Markers in Blood Serum

**Fig. 7.**
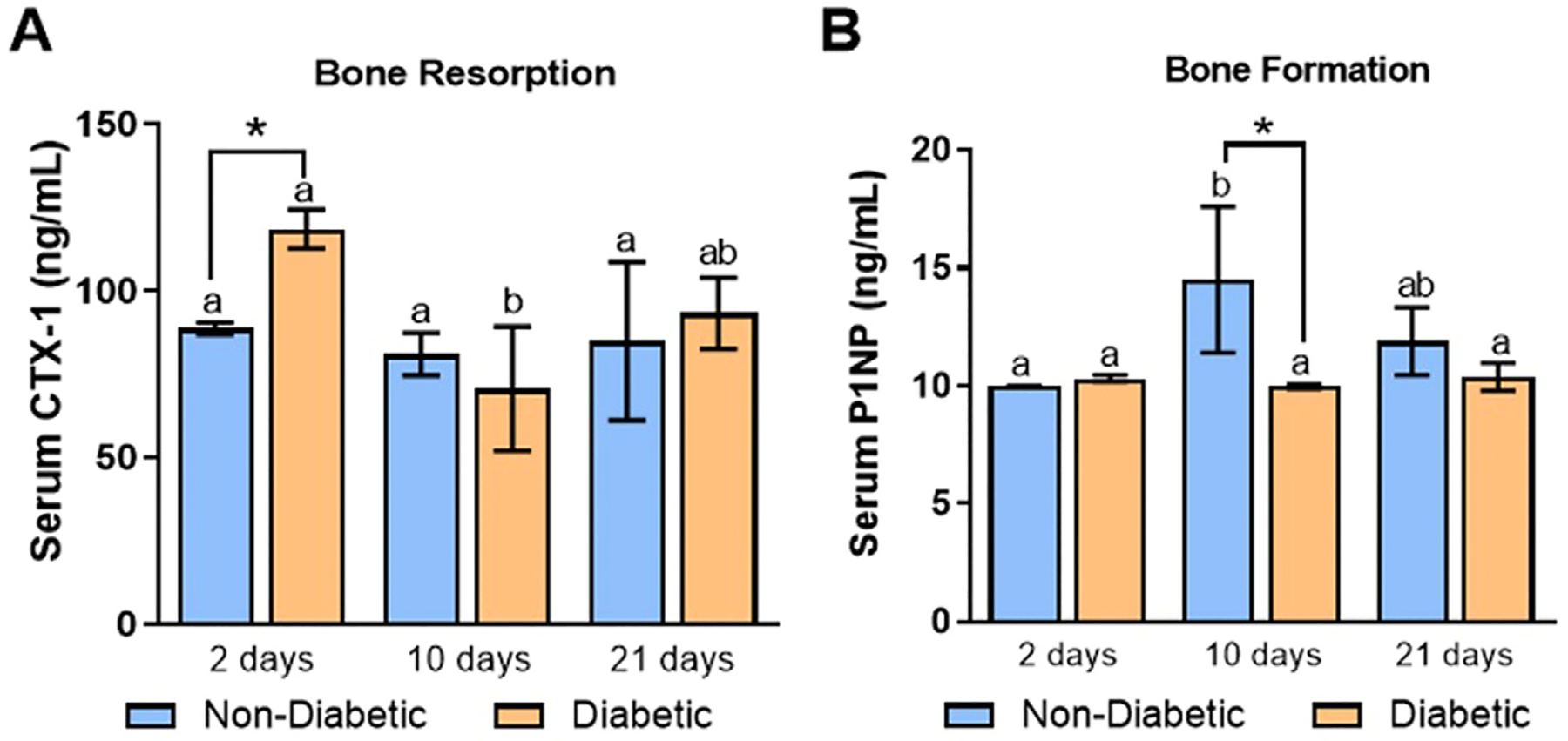
Serum levels of bone turnover markers in ND and D rats. ELISAs were used to determine the levels of the bone resorption marker, CTX-1 (*A*), and the bone formation marker, P1NP (*B*), at 2-, 10-, and 21-day postimplant placement. The mean ± SD are depicted. Symbol * indicates statistical significance between systemic conditions (ND or D; n = 5) within the same time point (*p* < .05). Letters *a* and *b* indicate statistical significance between time points within the same systemic conditions (a: *p* ≤ .05 and b: *p* ≤ .01).

Bone resorption marker CTX-1 was highest for the D group at 2 days (118.60 ± 5.82 ng/mL) and was significantly different from the ND group (88.78 ± 1.80 ng/mL) at the same time point. Both D and ND rats demonstrated a drop in bone resorption at 10 days with an increased trend at 21 days. P1NP, an indicator of bone formation, was significantly elevated for the ND group (14.49 ± 3.09 ng/mL) compared to the D group (9.98 ± 0.09 ng/mL) at 10 days. P1NP levels showed a plateaued trend over time, while the ND group resulted in an increase at 10 days and descended at 21 days. Values for blood serum bone turnover markers are shown in Fig. 7.

## Discussion

The aim of this study was to create and standardize an animal ORIF model for the future development of biomaterials used in high-risk cases, such as diabetic ankle fractures. To the best of our knowledge, this is the first study that assessed both healing and osseointegration in D and ND rats using an ORIF model. Fracture healing and osseointegration results demonstrated delayed healing in D animals compared to ND animals, which is in agreement with literature (17,18). This approach can be beneficial in the design of new implant surface treatments, materials and geometries.

Fracture repair and remodeling is a complex mechanism resembling endochondral ossification that involves several stages and biological processes, which include inflammation, soft callous formation, hard callous formation, and bone remodeling (15). Considering ORIF procedures where there is absolute stability, primary bone healing occurs due to compression fixation, without bone callus formation due to the lack of micromotion. When fractures are not fixated or have relative fixation, micromotion at the fracture site creates a bone callus during the healing process, thus leading to secondary bone healing (19). During secondary healing, inflammation is typically an acute response to injury of the tissues, where there is an interruption of vascular function, as well as deformities in overall structures that promote the secretion of platelets, macrophages, cytokines, growth factors, as well as early promotion of MSCs (15). Thereafter, soft callous formation and endochondral ossification is synthesized into a cartilage-like matrix that serves as a scaffold for hard callous formation.

In our study, the ORIF procedure (Fig. 1) resulted in a consistent and reproducible micro-fracture defect using a circular saw. As seen through microCT, there was significantly more fracture closure in ND rats at 21 days (Fig. 2). Histological evaluations demonstrated fracture healing (Fig. 3) and osseointegration at the implant site (Fig. 4A, C) over time. New bone in the fracture area for ND animals at 10 days, that wasn’t present for the D animals until the 21-day time point, is in accordance with previous studies where hypertrophic chondrocytes are shown to be present at 2 weeks (20). These results also correlated with microCT images (Fig. 2A), demonstrating the presence of the fracture at 21 days for the D group, while hyperdense bone was observed in the fracture area for the ND group. This observation with cancellous bone formation has previously been seen in rats after 3 weeks of healing (20).

During hard callous formation, higher levels of osteoblast activity represent the most active osteogenesis throughout the healing process. Osteoclast activity then demineralizes bone matrix to be turned into cortical bone in the remodeling phase, which resembles pre-injury structures. In each of these stages, there are unique histological characteristics that share common traits in both human and murine models (15). In our study, PCNA (cell proliferation) and RUNX2 (osteoblast differentiation) positive cells significantly increased at 10 days (Fig. 5), which corresponded to the early bone formation seen for ND animals. Bone formation and resorption trends are likely representative of endochondral ossification events (21).

For osseointegration of tissues surrounding the screw areas near the cortical region, increased BIC % at 10 days for the ND group was indicative of initial stabilization of bone matrix onto the implant surface and osseointegration of the cortical region (Fig. 4B). There was significantly more bone formation at 21 days in the implant area of the ND group, which also resulted in more positively stained PCNA+ and Runx2+ cells throughout each time point for ND animals (Fig. 6A, B). The reduced PCNA+ and Runx2+ cells in diabetic tissues further indicated impaired osteoblast proliferation.

A partly oxidized version of HMGB1, named disulfide HMGB1, has been implied to play a detrimental role on diabetic fracture healing (22,23). The area surrounding the implant resulted in increased quantities HMGB1 at 10- and 21-days (Fig. 6B) in the D group, and at all time points in the fracture area (Fig. 5B). HMGB1+ cells increased in the fracture area for the ND group at 10 days and subsided at 21 days. Interestingly, the D group resulted in significantly increased HMGB1 at 10 days that did not resolve at 21 days, while the ND group did resolve (Fig. 6B). Considering that diabetic patients normally have chronic inflammation, higher HMGB1 likely correlated with chronic inflammatory events in D animals.

We also evaluated systemic levels of bone turnover markers using blood plasma of ND and D animals. Interestingly, significant bone resorption at 2 days likely resulted in activated osteoclasts related to elevated inflammatory responses already present in D animals (Fig. 7A) (24,25). Collagen formation peaked at 10 days for ND animals and got mineralized at 21 days (Fig. 7B), which can clinically be correlated with histomorphometric bone formation (26,27).

This rodent ORIF model is not a direct replica of ORIF procedures seen in humans due to differences in gait patterns and mechanical loading; however, many physiological healing processes can be translated into a human model (28,29). It is important to note that the scientific rigor and reproducibility of this model provides clinical significance in terms of evaluating new surface approaches or drug delivery treatments. Considering that the systematic presence of diabetes significantly increases the likelihood of complications such as infection, malunion, nonunion and re-operation in a variety of surgically treated lower extremity fractures (6,17,18), it becomes crucial to engineer surface approaches that can be applied in nonoptimal environments. Thus, this model can provide prognostic information about osseous healing in response to novel orthopedic surface approaches and may aid in guiding future approaches in immunocompromised scenarios.

As with any in vivo study, ours had limitations. It is difficult to ensure that the comorbidities and complications associated with diabetes were congruently replicated. All experiments were conducted within 30 days of STZ administration, maintaining the time frame for the rats to become fully diabetic (7 days) before proceeding with the surgeries, as well as the longest time point for screw implantation (21 days). We performed a pilot in which animals were kept diabetic for 35 days, and they started to develop blindness, diarrhea, and incremental changes with normal behavior as time progressed. Considering this, we kept 30 days as the maximum time point to be diabetic. However, 21 days only covers bony callus formation in rodents (20). Longer time points (>6 weeks for complete fracture healing in normoglycemic rodents (20)) would allow the remodeling phase of bone healing to be assessed and likely show the effects of uncontrolled diabetes in osseous healing rates. Thus, increasing successive time points would give better clarity on trends seen in bone formation, remodeling, or degeneration in response to diabetes. Future studies should also incorporate both controlled and uncontrolled diabetes, where insulin is administered to appropriately represent the diabetic population.

In conclusion, within the limitations of the present study, this study demonstrated the feasibility of an ORIF model in rats, with distinct differences in fracture healing and osseointegration between diabetic and nondiabetic rats. Future studies should focus on longer time points or genetically diabetic animal models to fully understand the impacts of comorbidities and complications. This research offers important clinical implications for surgeons, emphasizing the need for personalized treatment strategies to improve healing outcomes in patients with diabetes undergoing ORIF procedures. Further studies that build upon these findings will help refine our understanding of these complex processes and pave the way for more effective treatments in the future.

## References

[1] Kaur M, Singh K. Review on titanium and titanium based alloys as biomaterials for orthopaedic applications. Mater Sci Eng C 2019;102:844–862. 10.1016/j.msec.2019.04.064 31147056

[2] Tengvall P, Lundström I. Physico-chemical considerations of titanium as a biomaterial. Clin Mater 1992;9:115–134. 10171197 10.1016/0267-6605(92)90056-y

[3] Melton LJ, Leibson CL, Achenbach SJ, Therneau TM, Khosla S. Fracture risk in type 2 diabetes: update of a population-based study. J Bone Miner Res 2008;23:1334–1342. 18348689 10.1359/JBMR.080323 PMC2574704

[4] Wukich DK, Kline AJ. The management of ankle fractures in patients with diabetes. J Bone Joint Surg Am 2008;90:1570–1578. 18594108 10.2106/JBJS.G.01673

[5] Jones KB, Maiers-Yelden KA, Marsh JL, Zimmerman MB, Estin M, Saltzman CL. Ankle fractures in patients with diabetes mellitus. J Bone Joint Surg Br 2005;87:489–495. 15795198 10.1302/0301-620X.87B4.15724

[6] Shibuya N, Humphers JM, Fluhman BL, Jupiter DC. Factors associated with nonunion, delayed union, and malunion in foot and ankle surgery in diabetic patients. J Foot Ankle Surg 2013;52:207–211. 23313498 10.1053/j.jfas.2012.11.012

[7] Zinman B, Haffner SM, Herman WH, Holman RR, Lachin JM, Kravitz BG, Paul G, Jones NP, Aftring RP, Viberti G, Kahn SE. Effect of rosiglitazone, metformin, and glyburide on bone biomarkers in patients with type 2 diabetes. J Clin Endocrinol Metab 2010;95:134–142. 19875477 10.1210/jc.2009-0572

[8] Manway JM, Blazek CD, Burns PR. Special considerations in the management of diabetic ankle fractures. Curr Rev Musculoskelet Med 2018;11:445–455. 29974403 10.1007/s12178-018-9508-x PMC6105492

[9] Shen H, Gardner AM, Vyas J, Ishida R, Tawfik VL. Modeling complex orthopedic trauma in rodents: bone, muscle and nerve injury and healing. Front Pharmacol 2020;11:1–7. 33597884 10.3389/fphar.2020.620485 PMC7882733

[10] Schindeler A, Mills RJ, Bobyn JD, Little DG. Preclinical models for orthopedic research and bone tissue engineering. J Orthop Res 2018;36:832–840. 29205478 10.1002/jor.23824

[11] Fajardo RJ, Karim L, Calley VI, Bouxsein ML. A review of rodent models of type 2 diabetic skeletal fragility. J Bone Miner Res 2014;29:1025–1040. 24585709 10.1002/jbmr.2210 PMC5315418

[12] Biguetti CC, De Oliva AH, Healy K, Mahmoud RH, Custodio IDC, Constantino DH, Ervolino E, Duarte MAH, Fakhouri WD, Matsumoto MA. Medication-related osteonecrosis of the jaws after tooth extraction in senescent female mice treated with zoledronic acid: microtomographic, histological and immunohistochemical characterization. PLoS One 2019;14:e0214173. 31199812 10.1371/journal.pone.0214173 PMC6568384

[13] Wheelis SE, Natarajan SA, Lakkasetter Chandrashekar B, Arteaga A, Garlet GP, Rodrigues DC. Effects of dicationic imidazolium-based ionic liquids on oral osseointegration of titanium implants: an in vivo biocompatibility study in multiple rat demographics. Genes (Basel) 2022;13:1–25. 10.3390/genes13040642 PMC9026960 35456448

[14] Biguetti CC, Cavalla F, Fonseca AC, Tabanez AP, Siddiqui DA, Wheelis SE, Taga R, Fakhouri WD, Silva RM, Rodrigues DC, Garlet GP. Effects of titanium corrosion products on in vivo biological response: a basis for the understanding of osseointegration failures mechanisms. Front Mater 2021;8:159.

[15] Wheelis SE, Biguetti CC, Natarajan S, Guida L, Hedden B, Garlet GP, Rodrigues DC. Investigation of the early healing response to dicationic imidazolium-based ionic liquids: a biocompatible coating for titanium implants. ACS Biomater Sci Eng 2020;6:984–994. 32656316 10.1021/acsbiomaterials.9b01884 PMC7351359

[16] Sotocinal SG, Sorge RE, Zaloum A, Tuttle AH, Martin LJ, Wieskopf JS, Mapplebeck JCS, Wei P, Zhan S, Zhang S, McDoougall JJ, King OD, Mogil JS. The Rat Grimace Scale: fautomated method for quantifying pain in the laboratory rat via facial expressions. Mol Pain 2011;7:55. 21801409 10.1186/1744-8069-7-55 PMC3163602

[17] Gortler H, Rusyn J, Godbout C, Chahal J, Schemitsch EH, Nauth A. Diabetes and healing outcomes in lower extremity fractures: a systematic review. Injury 2018;49:177–183. 29162268 10.1016/j.injury.2017.11.006

[18] Jupiter DC, Hsu ES, Liu GT, Reilly JG, Shibuya N. Risk factors for short-term complication after open reduction and internal fixation of ankle fractures: analysis of a large insurance claims database. J Foot Ankle Surg 2020;59:239–245. 32130984 10.1053/j.jfas.2019.08.003

[19] Sheen JR, Garla VV. Fracture healing overview. StatPearls. 2022. Available at: https://www.ncbi.nlm.nih.gov/books/NBK551678/. Accessed October 25, 2022. 31869142

[20] Handool KO, Ibrahim SM, Kaka U, Omar MA, Abu J, Yusoff MSM, Yusof LM. Optimization of a closed rat tibial fracture model. J Exp Orthop 2018;5:13–22. 29721763 10.1186/s40634-018-0128-6 PMC5931953

[21] Iwaki A, Jingushi S, Oda Y, Izumi T, Shida J, Tsuneyoshi M, Sugioka Y. Localization and quantification of proliferating cells during rat fracture repair: detection of proliferating cell nuclear antigen by immunohistochemistry. J Bone Miner Res 1997;12:96–102. 9240731 10.1359/jbmr.1997.12.1.96

[22] Muire PJ, Avila JJ, Lofgren AL, Wenke JC. Neutralization of HMGB1 improves fracture healing and *γδ* T lymphocyte counts at the fracture site in a polytrauma rat model. J Exp Orthop 2022;9:1–12. 35229226 10.1186/s40634-022-00453-3 PMC8885932

[23] Giacco F, Brownlee M. Oxidative stress and diabetic complications. Circ Res 2010;107:1058–1070. 21030723 10.1161/CIRCRESAHA.110.223545 PMC2996922

[24] Hamann C, Rauner M, Höhna Y, Bernhardt R, Mettelsiefen J, Goettsch C, Gunther K, Stolina M, Han C, Asuncion FJ, Ominsky MS, Hofbauer LC. Sclerostin antibody treatment improves bone mass, bone strength, and bone defect regeneration in rats with type 2 diabetes mellitus. J Bone Miner Res 2013;28:627–638. 23109114 10.1002/jbmr.1803

[25] Yu MD, Su BH, Zhang XX. Morphologic and molecular alteration during tibia fracture healing in rat. Eur Rev Med Pharmacol Sci 2018;22:1233–1240. 29565479 10.26355/eurrev_201803_14463

[26] Chavassieux P, Portero-Muzy N, Roux JP, Garnero P, Chapurlat R. Are biochemical markers of bone turnover representative of bone histomorphometry in 370 postmenopausal women? J Clin Endocrinol Metab 2015;100:4662–4668. 26505821 10.1210/jc.2015-2957

[27] Szulc P, Naylor K, Hoyle NR, Eastell R, Leary ET. Use of CTX-I and PINP as bone turnover markers: National Bone Health Alliance recommendations to standardize sample handling and patient preparation to reduce pre-analytical variability. Osteoporosis Int 2017;28:2541–2556. 10.1007/s00198-017-4082-4 28631236

[28] Moran CJ, Ramesh A, Brama PAJ, O’byrne JM, O’brien FJ, Levingstone TJ. The benefits and limitations of animal models for translational research in cartilage repair. J Exp Orthop 2016;3:1–12. 26915001 10.1186/s40634-015-0037-x PMC4703594

[29] Chu CR, Szczodry M, Bruno S. Animal models for cartilage regeneration and repair. Tissue Eng Part B Rev 2010;16:105–115. 19831641 10.1089/ten.teb.2009.0452 PMC3121784

